# Exploring the Mycobacteriophage Metaproteome: Phage Genomics as an Educational Platform

**DOI:** 10.1371/journal.pgen.0020092

**Published:** 2006-06-09

**Authors:** Graham F Hatfull, Marisa L Pedulla, Deborah Jacobs-Sera, Pauline M Cichon, Amy Foley, Michael E Ford, Rebecca M Gonda, Jennifer M Houtz, Andrew J Hryckowian, Vanessa A Kelchner, Swathi Namburi, Kostandin V Pajcini, Mark G Popovich, Donald T Schleicher, Brian Z Simanek, Alexis L Smith, Gina M Zdanowicz, Vanaja Kumar, Craig L Peebles, William R Jacobs, Jeffrey G Lawrence, Roger W Hendrix

**Affiliations:** 1 Department of Biological Sciences, University of Pittsburgh, Pittsburgh, Pennsylvania, United States of America; 2 Pittsburgh Bacteriophage Institute, University of Pittsburgh, Pittsburgh, Pennsylvania, United States of America; 3 Hampton High School, Allison Park, Pennsylvania, United States of America; 4 Lincoln High School, Elwood City, Pennsylvania, United States of America; 5 Tuberculosis Research Center, ICMR, Chetpet, Chennai, India; 6 Department of Microbiology and Immunology, Albert Einstein College of Medicine, New York, New York, United States of America; 7 Howard Hughes Medical Institute, Albert Einstein College of Medicine, New York, New York, United States of America; The Institute for Genomic Research, United States of America

## Abstract

Bacteriophages are the most abundant forms of life in the biosphere and carry genomes characterized by high genetic diversity and mosaic architectures. The complete sequences of 30 mycobacteriophage genomes show them collectively to encode 101 tRNAs, three tmRNAs, and 3,357 proteins belonging to 1,536 “phamilies” of related sequences, and a statistical analysis predicts that these represent approximately 50% of the total number of phamilies in the mycobacteriophage population. These phamilies contain 2.19 proteins on average; more than half (774) of them contain just a single protein sequence. Only six phamilies have representatives in more than half of the 30 genomes, and only three—encoding tape-measure proteins, lysins, and minor tail proteins—are present in all 30 phages, although these phamilies are themselves highly modular, such that no single amino acid sequence element is present in all 30 mycobacteriophage genomes. Of the 1,536 phamilies, only 230 (15%) have amino acid sequence similarity to previously reported proteins, reflecting the enormous genetic diversity of the entire phage population. The abundance and diversity of phages, the simplicity of phage isolation, and the relatively small size of phage genomes support bacteriophage isolation and comparative genomic analysis as a highly suitable platform for discovery-based education.

## Synopsis

Bacteriophages are viruses that infect bacterial hosts and are estimated to be the most numerous biological entities in the biosphere. Insights into the genetic diversity of the bacteriophage population and the evolutionary mechanisms that give rise to it can be obtained using comparative genomic analyses. The genomic analysis of 30 complete mycobacteriophages—viruses that infect mycobacterial hosts—reveals them to be genetically diverse and to contain many previously unidentified genes. The high diversity and relatively small genome sizes of these phages provide an ideal platform for introducing high school and undergraduate students to the research laboratory, isolating and naming novel viruses, and determining their genomic sequences. The thrill of discovering new viruses and previously unidentified genes, coupled with ownership of individual phage projects, provides strong motivations for students to engage in and pursue scientific research.

## Introduction

Approximately 10^31^ tailed bacteriophages are estimated to be on planet Earth, representing the majority of all biological entities in the biosphere [1,2]. Phages exert considerable influence on the microbial community [3] and cohabit with their bacterial hosts in highly dynamic relationships [3–5]. While phages show only somewhat limited morphological variation, the genomes of the approximately 300 completely sequenced double-strand DNA (dsDNA) phage genomes reveal both a high level of genetic diversity and a high proportion of genes that are dissimilar to any that have been previously sequenced [6–10]. Furthermore, phage genomic architecture is characterized by a high degree of mosaicism that likely arises from extensive horizontal genetic exchange occurring over perhaps as many as 3 billion years [9, 11–13].

dsDNA tailed phages are a substantial proportion of the total phage population, and the number of completely sequenced genomes has grown dramatically over the past 5 years to more than 150. These infect a wide variety of bacterial hosts, but the phages of enteric bacteria, bacteria relevant to the dairy industry, Mycobacterium spp., Pseudomomas spp., Staphylococcus spp., and marine bacteria are prevalent among the completely sequenced dsDNA tailed phage genomes [6,14–17]. These genomes vary in size from 25 to greater than 200 kilobase-pairs (kbp) and are harbored by viruses with a variety of virion morphologies [18]. The high degree of phage genetic diversity is indicated not only by the sequenced genomes of individual phages but also by viral metagenomic approaches showing that the majority of genes sequenced from uncultured viral libraries have no significant similarity to known genes [19–23].

Mycobacteriophages—viruses of the mycobacteria—have facilitated the development of mycobacterial genetic systems [24–27] and provided insights into viral diversity and the evolutionary mechanisms that generate them [6,28–31]. The comparative genomic analysis of 14 mycobacteriophages showed that they have relatively large genomes (average length, 70 kbp), contain large numbers of previously unidentified genes, and are highly diverse at both the nucleotide and amino acid sequence levels [6]. Moreover, phage genome architectures are pervasively mosaic; each genome apparently contains a unique combination of individual modules, of which the majority correspond to one or a small cluster of genes. Generation of these mosaic genomes reflects a high level of horizontal genetic exchange within the phage population, with illegitimate recombination events underlying the generation of new module boundaries. This model is supported by the identification of a small number of exchange events that have occurred relatively recently and for which there is no evidence of targeting to specific locations, such as gene extremities [6,7].

Since the majority of newly sequenced phage genes, including those from mycobacteriophages, are not closely related to known gene sequences, their functions remain largely unknown [15,19]. In many, but not all, dsDNA tailed phages, the genes encoding the virion structure and assembly functions are clustered into long operons with a well-defined gene order, and these genes can thus be more easily identified [32]. In phages adorned with a long flexible tail, the length of the tail is determined by the *tmp* gene, whose length corresponds directly to the tail length [6,33]. Since it is common for these phages to have tail lengths in excess of 100 nm, the *tmp* gene is frequently the longest open reading frame in the genome [6]. Outside of the structural operons, gene functions are well understood only in those phages that have been thoroughly dissected genetically and biochemically, and many phages contain large numbers of relatively small open reading frames of unknown function [6,34].

While the characteristic mosaic architecture of phage genomes can be explained by abundant horizontal genetic exchange events in their evolutionary history, little is known about which genomes participate in these events or the rates at which exchange occurs. Phages infecting the same host are expected to be more likely to exchange genes, but phages can readily switch or expand their host-range by a variety of mechanisms [35,36]. Moreover, some phages have very broad host-ranges [37]. In addition, capsid packaging imposes nonselective constraints on genome length that are quite distinct from the functional advantages and disadvantages of phage genes. Little is known about the rates of nonselective acquisition, or the subsequent fates of such genes in the phage population, although it is likely that gene acquisition and loss contribute significantly to the evolution of bacterial genomes [38–40].

Bacteriophages exchange genes not only among themselves but also with their host chromosomes, and it is common for phages to carry genes that profoundly influence the physiology of their hosts [41–44]; these include genes that confer bacterial pathogenicity, such as the toxins responsible for pathogenesis of cholera, diphtheria, and shigellosis [45,46]. If bacteriophage mosaicism is largely generated by a process of illegitimate recombination, then the acquisition of genes from the host is not surprising [6]. However, for host-like genes identified by phage genomics, it is unclear what advantage such genes may confer upon lysogenic hosts or whether they remain in the phage population for long or short periods.

We have expanded our collection of complete mycobacteriophage genomes to a total of 30 and utilized the enlarged set of gene sequences to explore their genetic diversity. The organization of protein-encoding genes into “phamilies” of related sequences provides insights into which genes are most prevalent in these phages, whether there are signature sequences that are characteristic of mycobacteriophages, and how phamily size corresponds to phage and bacterial homologs outside of the mycobacteriophage group. The genetic diversity, abundance of novel genes, and technical feasibility of phage isolation and genomic characterization strongly support the utilization of phage discovery and comparative genomics as an effective educational platform for undergraduate and high school students.

## Results/Discussion

### Isolation and Characterization of Mycobacteriophages

We previously reported the genome sequences of mycobacteriophages L5 [28], D29 [30], TM4 [31], and Bxb1 [29], along with a comparative genomic analysis of these and ten additional mycobacteriophages: Barnyard, Bxz1, Bxz2, Che8, Che9c, Che9d, Cjw1, Corndog, Omega, and Rosebush [6]. We have isolated an additional 16 mycobacteriophages using the methods described previously [6] from a variety of sources; Che12 was isolated in Chennai, India; Bethlehem and U2 are from Bethlehem, Pennsylvania, United States; 244 is from Connecticut, United States; and Catera, Cooper, Halo, Llij, Orion, PBI1, PG1, Pipefish, P-Lot, PMC, Qyrzula, and Wildcat are from Pittsburgh, Pennsylvania, United States, and the surrounding areas. Each of these 16 new phages was plaque-purified, grown in quantity, banded through an equilibrium CsCl density gradient, and used to isolate virion DNA. DNA was hydrodynamically sheared, cloned into plasmid vectors, sequenced, and assembled as described previously [6]. Many of these phages were isolated, named, and characterized by undergraduate and high school students, taking advantage of phage discovery and genomics as a platform for integrating scientific research and education as described in detail below.

### Mycobacteriophage Genometrics

Our view of the general features of mycobacteriophage genomes changes only modestly with a doubling of the number of genome sequences available (Table 1). The total sequence information is increased from 979,434 bp to 2,071,001 bp but the average genome length has not changed significantly. The largest of the newer genomes is Catera (153.7 kbp), slightly smaller than Bxz1 (156.1 kbp), the largest previously sequenced mycobacteriophage. One of the newly sequenced genomes, Halo, is only 42.3 kbp long, substantially smaller than any other mycobacteriophage genome (Table 1). Neither the base composition (average %GC content, 63.7%) nor the average ORF length (600 bp) has changed significantly (Table 1). These genomes also encode 101 tRNAs and three tmRNAs. The distribution of these small translation-system RNAs is quite uneven, with three phages (Bxz1, Wildcat, and Catera) contributing 80% of the total tRNAs and all three tmRNAs. Since the primary focus of this paper is to report on the diversity of mycobacteriophage gene phamilies and the use of phage genomics as a discovery-based educational platform, further details of the individual phages and their genomes will be reported elsewhere.

**Table 1:**
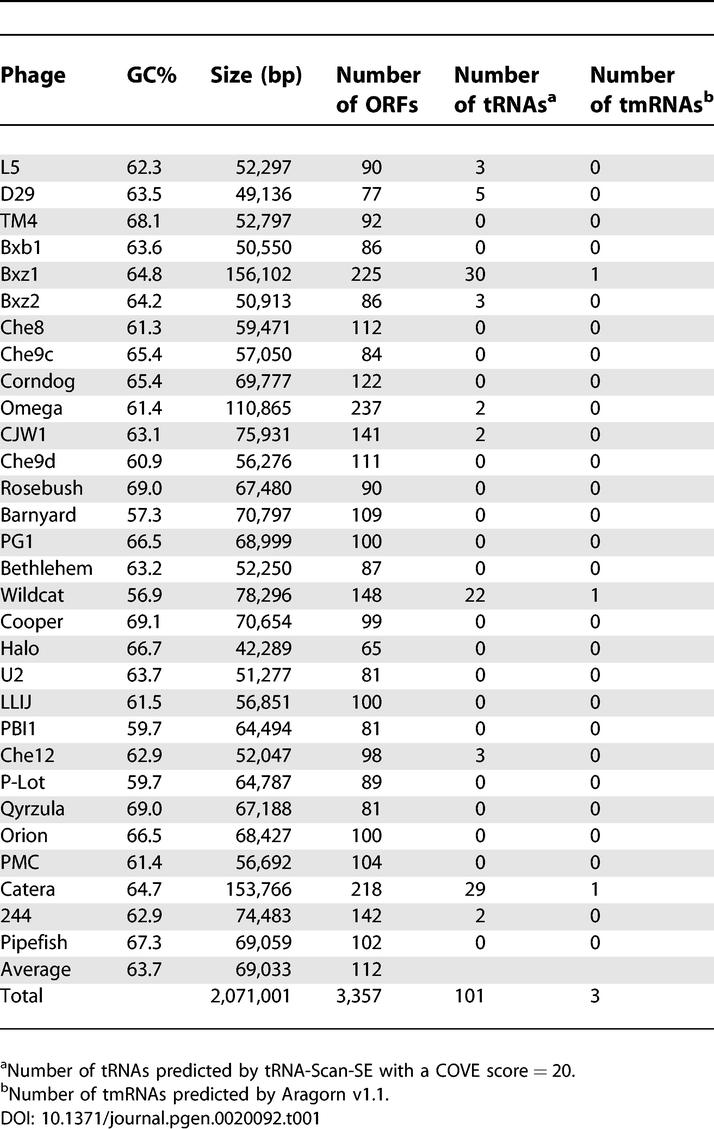
Features of Completely Sequenced Mycobacteriophage Genomes

### Nucleotide Sequence Diversity of Mycobacteriophages

**Figure 1:**
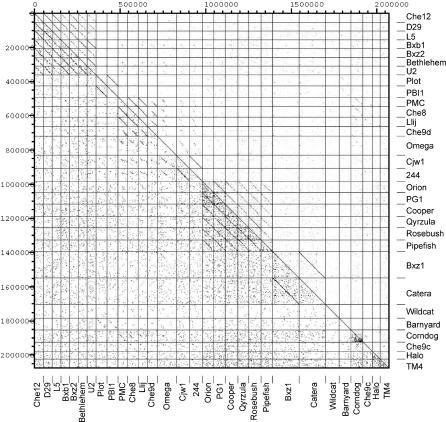
Nucleotide Sequence Comparison of 30 Mycobacteriophage Genomes as Illustrated in a Dotter Plot Using a Sliding Window of 25 bp [63] The lower triangle represents the relationships at an elevated level of gray-scale relative to the upper triangle, revealing weaker sequence relationships.

Comparison of the 30 mycobacteriophages with each other at the nucleotide level reveals considerable overall diversity, with small groups having recognizable sequence similarity (Figure 1). The most numerous phage genome cluster contains seven that are more closely related to each other than to other phages; these include L5, the first sequenced mycobacteriophage genome [28], D29 [30], Bxb1 [29], Bxz2 [6], Che12, Bethlehem, and U2. The next most numerous group contains six members, including the previously described Rosebush [6], Orion, PG1, Cooper, Qyrzula, and Pipefish. Phages PMC, Che8, and Llij form another cluster, with parts of Che9d and Omega having similarity to these; two-member groups are formed by phages P-Lot and PBI1, Cjw1 and 244, and Bxz1 and Catera. Phages showing little or no nucleotide sequence similarity to any of the others are TM4, Halo, Che9c, Barnyard, Corndog, and Wildcat.

The profile of nucleotide sequence similarities differs in several notable ways from the previously reported comparison of only 14 genomes [6]. While the overall diversity remains very high, 11 of the newly sequenced genomes have recognizable nucleotide sequence similarity to at least one of the previously reported genomes. A particular surprise is the finding that five of the newly sequenced genomes have detectable nucleotide sequence similarity to the previously sequenced genome of phage Rosebush (of which no closely related genomes had been described), although, with the exception of Qyrzula, the degree of similarity is low. In contrast, the Bxz1/Catera and Cjw1/244 groups are more closely related than any other pairs of mycobacteriophage genomes. Both pairs exhibit greater than 90% nucleotide sequence identity with a number of small insertions or deletions accounting for much of the difference. In contrast to other groups of phages that have been analyzed, the diversity at the nucleotide sequence level of the mycobacteriophages appears to be greater than that of either dairy phages [14] or staphylococcal phages [17], although it is not yet clear whether this reflects underlying biological diversity differences or just the relatively small numbers that have been analyzed and the isolation approaches utilized.

There is no clear correlation between membership in these clusters of similar phages and the phages' geographic origins. Although all the members of the Rosebush group came from the environs of Pittsburgh, the seven members of the L5 cluster came from Japan, New York City, California, Chenai, India, and Bethlehem, PA. The smaller groups are also mostly geographically diverse. Our view at the current level of data collection is that the clusters of similar phages are widely distributed geographically.

### Mycobacteriophage Genes and Gene Phamilies

**Figure 2:**
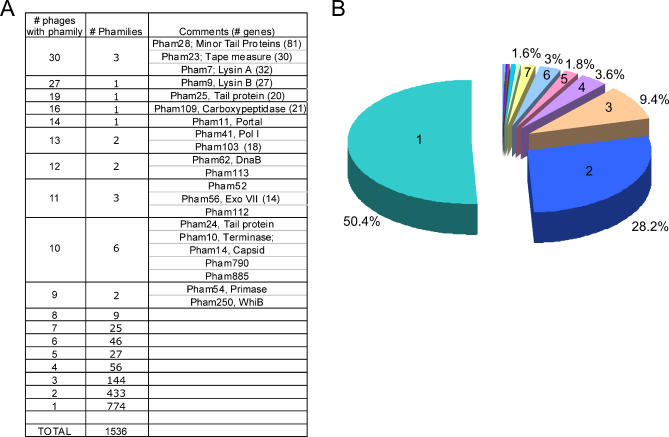
Size and Distribution of Mycobacteriophage Phamilies All 3,357 mycobacteriophage genes were assorted into 1,536 phamilies based on amino acid sequence similarity with a BLAST E value of 0.001 or better to at least one other member of the phamily. (A) The distribution of phamilies is shown ranked according to the number of mycobacteriophage genomes containing at least one phamily member. Examples of specific phams and the total number of mycobacteriophage genes within that pham are shown. (B) Pie-chart representation of the phamily-size distribution. Phamilies with eight or more members represent about 2% of the total.

The 30 mycobacteriophage genomes encode a total of 3,357 open reading frames (Table 1). As expected, the newly sequenced genomes possess a mosaic architecture similar to those described previously, with modules—frequently containing just a single gene—shared by otherwise distantly related phages. In order to better understand this genetic diversity, we have assembled all 3,357 open reading frames into gene phamilies, i.e., groups of related sequences, using the criteria that an encoded protein must share amino acid sequence similarity at an E-value of 0.001 or better or 25% amino acid identity across its length with at least one other member of the phamily (Table S1). This generates a total of 1,536 phamilies, with a mean phamily size of 2.19 genes (Figure 2). However, more than half of the phamilies (774, 50.3%) contain just a single constituent gene, and 88% of the genes are contained within phamilies containing three or fewer members (Figure 2).

**Figure 3:**
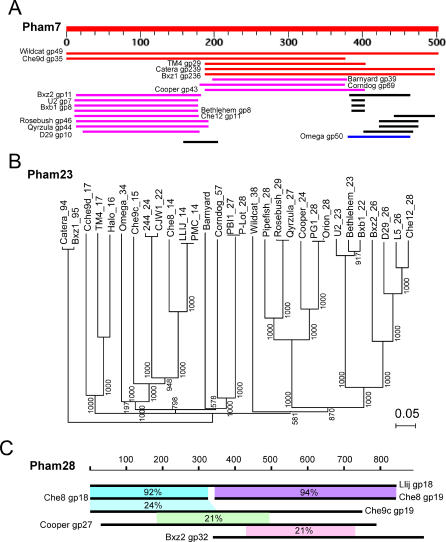
Complex Relationships within Highly Abundant Mycobacteriophage Phamilies (A) Complex relationships among members of the Pham7 (Lysin A) phamily. The output of a BLAST comparison of Wildcat gp49 against other mycobacteriophage proteins shows that only 16 other mycobacteriophage proteins are matched and that these correspond to different parts of Wildcat gp49. Colored bars represent the strength of the matches, with red being the strongest, followed by purple, blue, and black. (B) Phylogenetic relationships between members of mycobacteriophage Pham23 (tape-measure protein; Tmp). Amino acid sequences for each of the 30 constituent members of Pham23 were aligned using ClustalW and the unrooted phylogenetic relationships represented using NJTree. Bootstrap values from 1,000 reiterations are shown. (C) Chimerism in Pham28 (minor tail) proteins. Llij gp18 is related to both gp18 and gp19 of phage Che8 at high levels of amino acid sequence identity, and these proteins are related in turn to other members of Pham28 as shown.

Surprisingly, there are only three phamilies that contain members in all 30 of the mycobacteriophage genomes. Since these phamilies have the potential to contain genes or domains that are present in all mycobacteriophage genomes—and may thus correspond to mycobacteriophage signatures—we have examined these more closely. The first of these, Pham7, contains Lysin A genes, one of two putative lysins encoded by the mycobacteriophages [47]. The second lysin, Lysin B (Pham9), is also present in 27 of the 30 genomes (Figure 2); however, both of these phams are highly diverse, and they appear to be composed of subgenic modules with reasonably defined boundaries (Figure 3A). While each of the Pham7 members has sequence similarity to at least one other member of the phamily, no single sequence element within Pham7 is present in all 30 genomes (Figure 3A).

Both of the remaining 30-genome phamilies correspond to tail proteins. One of these encodes the tape-measure protein (Tmp) (Pham23) that plays a role in tail assembly and determines the lengths of noncontractile tails [6,33]. This phamily is also highly diverse, but the relationships among the members are complicated, and no well-defined boundaries between shared modules could be recognized; this complexity is illustrated by the long branch lengths within a phylogenetic analysis (Figures 3B). The second tail protein phamily (Pham28) is equally complicated, with ill-defined module boundaries (Figure 3C), and a total of 81 genes fall into this phamily (Figure 2A). Nevertheless, as seen in Pham7, no single sequence element in either of these tail protein phamilies is present in all 30 genomes.

It is noteworthy that the six most abundant phamilies (Phams 28, 23, 7, 9, 25, and 109; Figure 2A) appear to be highly represented not just because of the conservation of essential functions but also because of their highly divergent and modular natures. These phamilies correspond to phage functions—predominantly tail proteins and lysis proteins—that are expected to be intimately involved in interacting with their bacterial hosts, and high diversity among phage tail proteins has been described previously [48,49]. Although the Pham109 members are related to carboxypeptidases (Figure 2A), Pham109 genes are typically located among tail genes [6,29]. Thus, we postulate that they are likely to be structural components of tails.

### Phamily-Based Clustering of Mycobacteriophage Genomes

**Figure 4:**
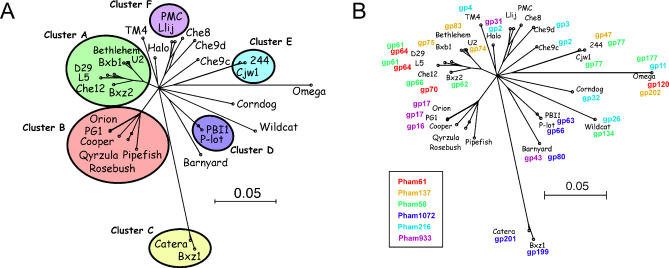
Representation of Mycobacteriophage Clusters Using Splitstree (A) The relationships between 30 mycobacteriophages are represented by Splitstree representation of a dataset in which each of the 1,536 gene phamilies is annotated as being either present or absent in each of the 30 genomes. Clusters A through F of genomes that are more closely-related to each other than to other mycobacteriophages are shown by colored circles. (B) The distribution of the members of six phamilies on the Splitstree representation in (A) illustrates that individual phamilies have notably different evolutionary histories than the aggregate representation.

The comparison of mycobacteriophage genomes at the nucleotide level (Figure 1) reveals not only considerable genetic diversity but also small groups of phages that appear to be more closely related to each other than they are to other mycobacteriophages. To explore this further, we examined the relationships among these phages by asking whether or not each genome contains a member of each gene phamily by using the program Splitstree [50], which accommodates alternative phylogenetic relationships, to express these data (Figure 4A). This analysis reveals six clearly defined groups of genomes that we have termed clusters A through F. Cluster A contains the previously characterized phages L5, D29, Bxb1, and Bxz2, plus the newly sequenced genomes of Bethlehem, Che12, and U2. The second most numerous cluster (cluster B) includes six genomes, of which only one (Rosebush) was previously characterized [6]; the remaining four clusters are closely related pairs of genomes (Figure 4A).

The relationships presented in Figure 4A help to organize the discussion of the general features of these genomes, and we note that this approach bears some resemblance to the phage proteomic tree described previously [51], although the Splitstree presentation enables at least partial inclusion of phylogenetic ambiguities that arise from the comparison of genomes that are pervasively mosaic in their architecture. But while these representations reflect the global relationships of the phages, it is important to note that they represent aggregate representations of the evolutionary histories of these viruses, and this ignores the numerous constituent phamilies that have phylogenies that are distinct from that shown in Figure 4A. The six phamilies (Phams 61, 137, 58, 1,072, 216, and 933) shown in Figure 4B illustrate this problem, underscoring the important role of horizontal genetic exchange in phage evolution.

**Figure 5:**
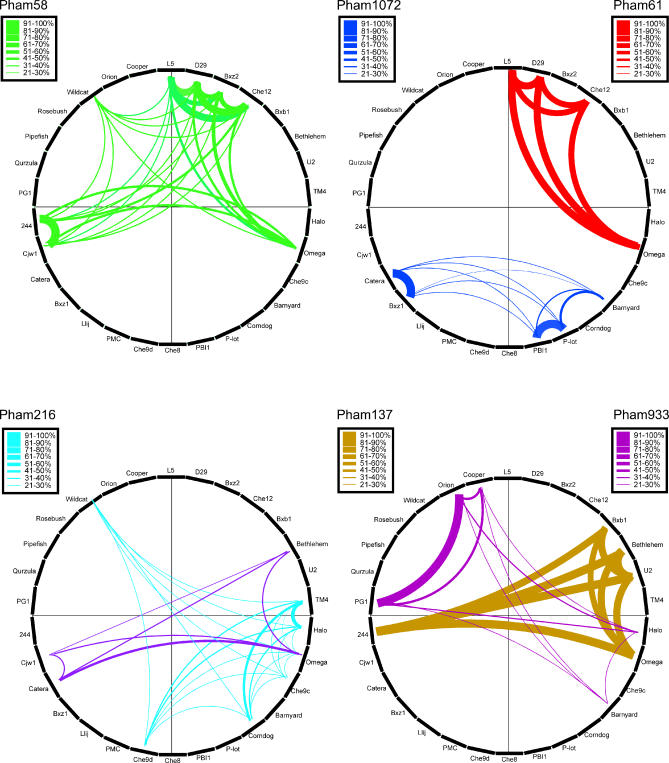
Phamily Circle Representations of Phamily Relationships All 30 genomes are shown around the circumference, and the phamily members are linked by a line with the width representing the degree of similarity. Phamily circles of Pham58 (upper left), Pham61 and Pham1072 (upper right), and Pham137 and Pham993 (lower right) are shown using different colors for different Phams. Pham216 (bottom left) is shown in turquoise, with the intein present within the Omega phamily member (which has a different set of relationships) shown in purple.

An alternative representation of phage genome relationships utilizes phamily circles to identify the participants and indicate the strength of relationships within each phamily (Figure 5). For example, Pham58 is present in eight genomes, with the strongest relationships between the phamily members in four of the seven components of cluster A and both members of cluster E. In contrast, Pham61 members are also in three of the same four members of cluster A as Pham58, but these are all closely related to a Pham61 member in Omega. This representation also illustrates the phylogeny of the intein present in the Pham216 member in Omega, which differs substantially from the remainder of the phamily members. While the phamily circles shown in Figure 5 obviously represent only a subset of all of the possible genetic relationships in the bacteriophages, a hypothetical extension to all of the 762 phamilies that contain two or more members would constitute a complete and accurate representation of the phylogenies of the protein-encoding genes of these genomes. The future development of automated circle drawing software should greatly facilitate this.

### How Many Mycobacteriophage Phams Exist?

**Figure 6:**
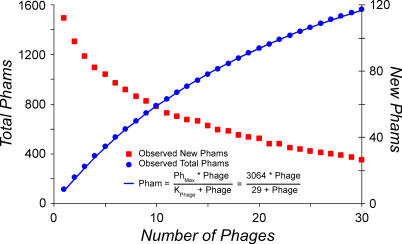
Estimating the Number of Mycobacteriophage Phams A subset of the 30 phages was randomly selected without replacement, and the total number of Phams was determined; this was repeated 10,000 times with the mean shown as a blue circle. For each subset, an additional phage was then randomly chosen, and the average number of new Phams found in that phage was determined; these data are shown as red squares. The total number of Phams was fit to a hyperbolic function, with the best-fit equation determined by least-squares regression.

To estimate the total number of phams shared among mycobacteriophages, we calculated the number of phams found among randomly chosen subsets of the 30 phages described above (Figure 6). A hyperbolic curve was fit to the data, where PhamMax is the maximum number of phams in the population being sampled, and K_Phage is the number of phages that must be sampled to uncover one-half of the phams. We found that a curve with PhamMax = 3,064 and K_Phage = 29 fit the data well (Figure 6). That is, this sample of 30 phages contains representatives of only half of the predicted total number of 3,064 phams to be found among mycobacteriophages, and sequencing a further 100 mycobacteriophage genomes would probably uncover members of an additional approximately 1,000 phams, assuming that phages that are closely related to known phages are not excluded from the sample. We note that on average the addition of a thirtieth phage to a randomly selected group of 29 phages leads to identifying approximately 25 new phams.

### Genetic Novelty of Phage Phamilies

**Figure 7:**
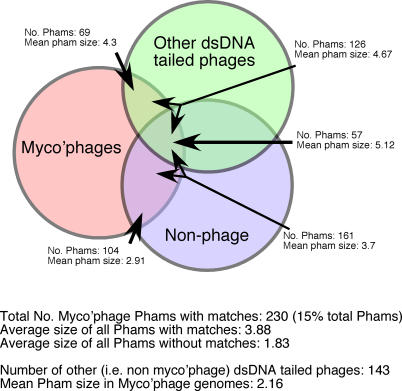
Relationships between Mycobacteriophage Phams and Previously Sequenced Proteins The number and size of mycobacteriophage phamilies with sequence similarity to nonmycobacteriophage genes are shown. The numbers of Phams shared by mycobacteriophages, other phages, and nonphage genomes are shown, along with the average pham size, defined as the number of mycobacteriophage genomes containing at least one member of that phamily. The red circle represents mycobacteriophage genomes, the green circle represents all dsDNA phage genomes other than the mycobacteriophages, and the blue circle represents all nonphage genomes. The number of phams shared between these groups and the mean mycobacteriophages pham size of those phams are shown, with arrows indicating whether they are shared by mycobacteriophages (red circle), nonmycobacteriophage phage genomes (green circle), or nonphage genomes (blue circle).

The organization of mycobacteriophage genes into phamilies simplifies the process of determining how these are related to genomes of other phages and their bacterial hosts. For example, of the 1,536 phamilies, only 230 (15%) have recognizable sequence similarity (Blast E value of 0.001 or better) to existing (nonmycobacteriophage) database entries (Figure 7). Remarkably, over 85% of these mycobacteriophage phamilies, a total of 1,306, represent previously unidentified genetic sequences, consistent with the idea that phages may represent the largest reservoir of unexplored genes in the biosphere [6, 19]. Interestingly, only 126 (54.8%) of the 230 phamilies with matches correspond to genes found in other phages, whereas 104 (45.2%) are related to nonphage (predominantly bacterial) genomes; 57 (24.8%) of the 230 phamilies are found in both phage and bacterial genomes (Figure 7). Some the phams listed as exclusively matching bacterial genomes may be identifying unrecognized or unannotated prophages.

It is clear from this analysis as well as that of other phage genomes that there is little overlap between the metaproteome of phages and that of prokaryotes. Moreover, typically, greater than 80% of genes within bacterial genomes can be assigned to clusters of orthologous groups, and in some cases (e.g., *Buchnera*) virtually every gene can [52, 53]; this is in contrast to the high proportion of mycobacteriophage genes (approximately 50%) that are unrelated to other genes (i.e., ORFans). Grouping of the phage genes into phamilies is therefore justified similarly to the separate ordering of eukaryotic genes into eukaryotic clusters of orthologous genes [53]. We also note that only a small proportion of mycobacteriophage phamilies (approximately 20%) would qualify as adding to or expanding the current cluster of orthologous groups database.

### Phamily Flux

Examination of the phams that match other phage or nonphage genes, or both, reveals qualitative differences among these groups, particularly in regard to the numbers of mycobacteriophage genomes within each pham. For example, the average pham size (defined as the number of mycobacteriophage genomes containing a phamily member) of all phams with matches to nonmycobacteriophage genes is 3.88, compared to 1.83 for those with no matches (Figure 7). Thus, if a mycobacteriophage gene has sequence similarity to any nonmycobacteriophage gene, it will have more relatives within the group of mycobacteriophages than one that does not.

The average pham size is different for the groups of phams that match phage and nonphage genes (Figure 7). However, the average pham size for the 126 phams matching all other phage genes is 4.67, substantially larger than the average size for all phams (2.19) or those with matches (3.88) (Figure 7); the subset of these that specifically matches both phage and nonphage sequences is even larger, with an average pham size of 5.12 (Figure 7). On the other hand, the average pham size for the 104 phams that exclusively match nonphage genes is only 2.91.

What is the basis for different distributions of pham sizes? While the dataset remains relatively small and the 30 genomes are not all equally different from each other (Figures 1 and 4), we postulate that the differences in average pham sizes reflect the extent to which these phams provide functions of general utility to the mycobacteriophages (i.e., high pham size) or provide specialist functions to smaller numbers of mycobacteriophages that are required to infect specific hosts to or survive within particular environmental or biological niches. Examples of the pham group that match other phages include both structure and assembly genes (e.g., capsids, portals, terminases) as well as nonstructural genes (e.g., *ruvC,* integrases, excises), but these phams presumably provide important functions to both mycobacteriophages and other phages. In contrast, the phams matching nonphage gene products with smaller pham sizes include gene products such as WhiB, Glutaredoxins, FtsK, Lsr2, DinD, DinG, Ro, and PurA; these presumably provide more specialist functions. It seems likely that these specialist functions have been acquired directly from host genomes, and they are relatively rare because of their restricted utility to a subset of phages, rather than their lack of opportunity to spread more broadly among this group of phages that infect at least one common host (i.e., Mycobacterium smegmatis).

### Constraints on the Modular Construction of Phage Genomes

If there are approximately 3,000 different sequence phamilies among phages that infect M. smegmatis, how many different ways can these be combined to make a functional phage genome? If we assume that an average genome contains 100 genes, then there are approximately 10^350^ possible ordered phamily combinations. Since there are estimated to be 10^31^ phage particles in the biosphere and mycobacteriophages represent only a fraction of these, it is clear that only a miniscule fraction of the conceivable gene combinations and organizations have been used. More important, we expect that there are strong functional constraints imposed on the generation of competent phage genomes. These constraints can be grouped into four main categories. First, each genome must encode all necessary functions not provided by the host, mandating inclusion of virion structural and assembly genes, lysis genes, and possibly some DNA replication functions. Thus, a specific subset of phamilies with required functions must be present in each genome. Second, specific gene combinations are required when a gene can provide functional benefits only in combination with another gene. One example might be the joint action of holin and lysin components of the lysis machinery. Third, some phamilies may encode alternative functions, where the inclusion of a member of one phamily (e.g., a virion capsid) precludes the inclusion of any members from another phamily (e.g., a nonhomologous capsid protein). Fourth, gene organization is likely to be an important constraint, for example, allowing cotranscription of genes that need to be coregulated.

Clearly, the 30 mycobacteriophage genomes are not simply the result of random assortment of phamilies. At both the nucleotide (Figure 1) and protein (Figure 4) levels, we can identify clusters of phages that exhibit greater levels of similarity to each other than to the larger population; for example, 21 of the 30 phages fall into six separate clusters at the nucleotide sequence and the phamily inclusion levels used here (Figure 4). While it is likely that this clustering reflects in part the particular host and the isolation procedures used, it also suggests that there are pools of related phages that enjoy success within the current environment. This is reminiscent of what has been seen for phages of other hosts, including those of *Escherichia coli,* where separate clusters are represented by phages lambda, T4, T7, and P2. The types of phages defined by the clusters of mycobacteriophages are not obviously correlated with the types seen in the coliphages, suggesting the possibility that each host type has a distinct set of phage types associated with it. We suspect instead that each of the clusters defined by the current collection of genome sequences is part of a continuum of types extending over a range of hosts, with no sharply definable boundaries. However, more data will be required to clarify this question.

It seems likely that an additional constraint on the assortment of phamilies is that their distribution within genomes is not independent of each other. For example, examination of the data set reveals that Pham56 is found in diverse bacteriophages but only in those that carry the Pham25 minor tail protein, suggesting both a role for Pham56 in virion assembly and a lack of function in genomes lacking Pham25. The nine members of Pham297 are not only found in Pham25-bearing genomes, but they are adjacent to the Pham25-encoding genes, suggesting an even more intimate association between these proteins. The coassociation of genes among bacteriophage genomes can only be assessed using a large collection of diverse genomes, and this demonstrates how datasets such as this one can be used to uncover plausible gene functions and interactions.

### Phage Discovery as an Educational Tool

The newly discovered phages described here were isolated and characterized by junior members of the laboratory, including high school and undergraduate students (Table 2). The educational benefits of performing scientific research have been reported previously [54,55], although identifying suitable research projects and laboratory environments represents a significant challenge to the research community. The Phagehunters Program developed at the University of Pittsburgh—in which students discover and genomically characterize their own bacteriophages—provides a particularly strong combination of attributes that maximize the educational benefits within a research environment; we note that a successful program with some common features also was developed by R. Young and colleagues [56]. While there may be many others, we have identified seven key attributes of this project that facilitate successful outcomes, and these are described in Table 3. Identifying the key features of projects that are well suited for student research should facilitate the identification and development of other effective programs.

**Table 2:**
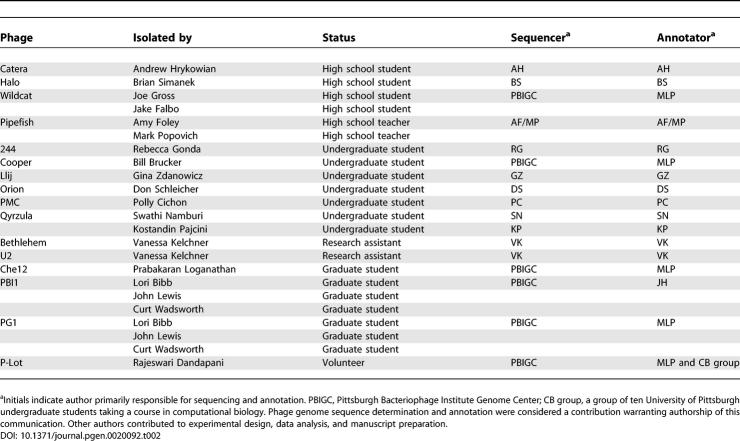
Isolation, Sequencing, and Annotation of Mycobacteriophage Genomes

**Table 3:**
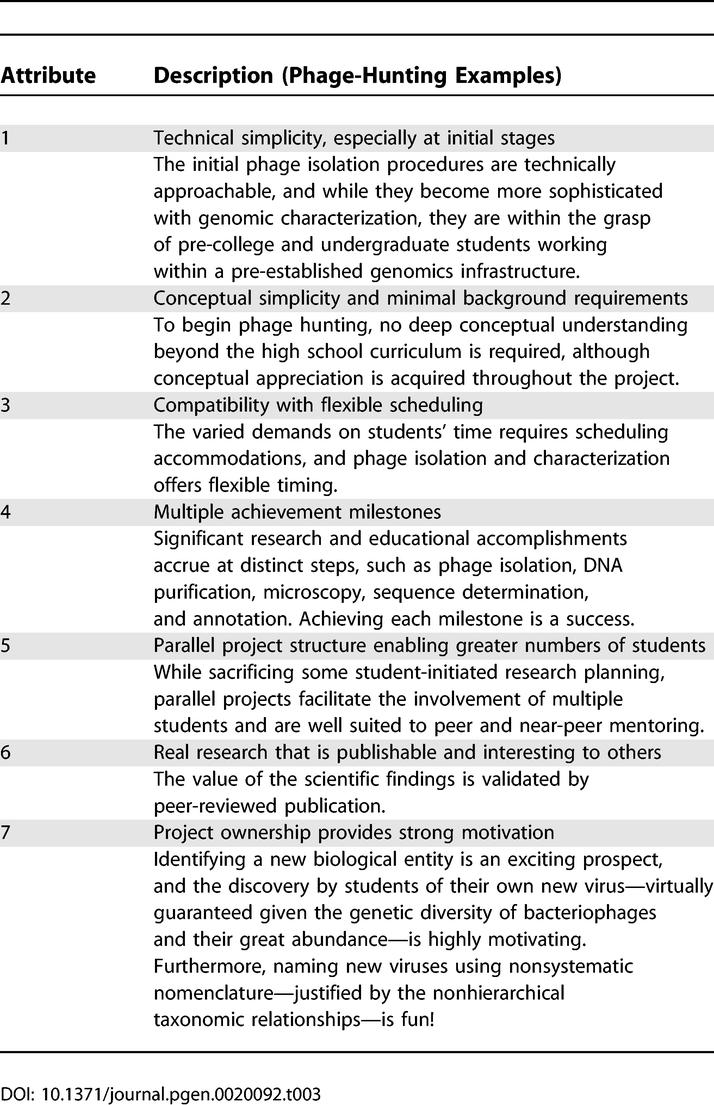
Seven Helpful Attributes for High School and Undergraduate Research Projects

Two particularly important features of this educational platform are the strong emphasis on scientific discovery and project ownership. As evident from the comparative genomic analysis described above, there are three main discovery elements. First, there is an excellent prospect of each student isolating a phage from the environment that is different to all previously described viruses. Second, within each genome there is an opportunity to discover new genes that are distinct to all previously identified genes. Third, many of these phage genomes surprisingly contain homologs of known genes that have not been previously found in phage genomes. The high diversity of the mycobacteriophage population (Figure 1), the preponderance of novel genes (Figure 7), and the mosaic architecture of these genomes provide a high promise of discovery for each participating student.

The opportunity for students to discover novel genes and viruses is important since it is stimulating and highly motivating, providing a strong impetus for students to become engaged in scientific research and to maintain their involvement even through the more challenging aspects of their projects. The modest genome size of the phages is such that a single student can reasonably manage an individual phage, and project ownership adds motivation and commitment. The prospect of naming their new phage generates considerable interest among these novice scientists while also offering opportunities to learn about the justification of a nonsystematic nomenclature for viruses that contain genomes with highly individualized mosaic architectures. This educational platform is clearly not restricted to mycobacteriophages and can be readily extended to the isolation and characterization of environmental phages for other nonpathogenic hosts; comparative genomic analyses of dairy, staphylococcal, and pseudomonal phages show that these also are genetically diverse and typically harbor a high proportion of novel genes [14,16,17].

The development of multiple projects with parallel structures as described here brings both advantages and disadvantages. The main disadvantage is that since the research path is well established, students generally have only limited opportunities for experimental planning and design. However, there are also considerable advantages to parallel project structures. First, it provides opportunities for a greater number of students to participate than if each were independently structured. Second, it facilitates the training of students through peer and near-peer mentoring systems, and we find that mentorship of high school students by undergraduates is a particularly effective combination. Moreover, undergraduate student mentors can be readily trained using the Entering Mentoring program developed by Pfund and colleagues [57]. The parallel project structure, coupled with this mentoring system and the technical simplicity of the initial project stages, represents essential ingredients for enabling students in the early stages of their educational development to engage in scientific research.

Finally, this phage discovery educational platform requires only modest prior comprehension of biological facts and concepts. This simplifies access of young students to scientific research and provides opportunities to students who do not necessarily excel in more traditional classroom settings. The platform offers numerous opportunities for students to learn concepts in microbiology, ecology, genetics, computational biology, and evolution within an inquiry-driven environment and is fully inclusive of a diverse variety of learning styles. Additionally, the significant bioinformatics component of the program appeals to students with computer science and engineering backgrounds, and in doing so, it creates a diverse research group that offers advantages both to the participants and the research agenda itself. Detailed protocols are available at http://www.pitt.edu/~gfh.

### Concluding Remarks

The comparative genomic analysis of 30 mycobacteriophage genomes provides important new insights into the diversity and architecture of phage genomes and offers insights about gene exchange between phage genomes and between phages and their hosts. It is likely that these general features will be shared by most other phages, and the recent comparative analysis of 27 *Staphylococcus* and 18 *Pseudomonas* phages also shows relatively high genetic diversity [16,17]. Phage isolation and genomics is a powerful educational platform that provides research opportunities to students from diverse educational backgrounds. The high diversity of the phage population offers the excitement that each student can isolate a unique virus and uses genomic approaches to understand the relationship of the newly discovered phage to the broader biological world. The ability of students to contribute successfully to achieving the key scientific goals of understanding viral diversity, and the underlying evolutionary mechanisms that give rise to it, suggests that phage isolation and characterization can be used broadly for educational purposes.

## Materials and Methods

### Phage isolation.

Phages were isolated from the following locations: Cooper, Halo, Llij, Orion, PBI1, PG1, P-Lot, Pipefish, PMC, and Qyrzula were from Pittsburgh; Bethlehem and U2, Bethlehem, Pennsylvania, Unites States; Catera and Wildcat, Latrobe, Pennsylvania, United States; 244, Connecticut; and Che12, Chennai, India. Samples from various sources were extracted with phage buffer, plated directly onto solid overlays containing 0.35% agar and Mycobacterium smegmatis mc^2^155, and incubated at 37 °C for 24 h as described previously, with the exception of Che12, which was isolated using Mycobacterium tuberculosis as a host [6]. Individual plaques were picked and purified through several rounds and purified by CsCl equilibrium density gradient centrifugation.

### Genome sequencing and analysis.

Approximately 10 μg of purified phage DNA was sheared hydrodynamically and repaired, and 1- to 3-kbp fragments inserted into plasmid pBluescript (Stratagene, La Jolla, California, United States). Individual clones were sequenced using ABI3730 or ABI3100 instruments (Applied Biosystems, Foster City, California, United States), and the sequences were assembled into a single or small number of contigs [58]. At approximately 8-fold redundancy, sequence data from oligonucleotide primers used with phage template DNA generated a single contig and resolved sequence ambiguities. Genome termini were sometimes identifiable as an overabundance of clone ends, and a comparison of the sequences generated using primers annealed to ligated and unligated phage DNA allowed determination of the molecular ends, where possible.

Sequence assembly was performed using the Phred/Phrap/Consed suite of programs and annotated using a variety of programs including DNA Master (available from http://cobamide2.bio.pitt.edu), Genemark [59], and Glimmer [60]. tRNA and tmRNA genes were identified using tRNA-Scan-SE and ARAGORN [61,62]. BLAST analyses were performed either locally or remotely at the National Center for Biotechnology Information and were used to assemble a database of related sequences in Microsoft Excel.

## Supporting Information

Table S1 The Pham Database Each of the putative proteins encoded by 30 mycobacteriophage genomes are tabulated according to sequence similarity and assembled into phamilies. Putative pham functions are also listed.
(250 KB XLS)

### Accession Numbers

GenBank (http://www.ncbi.nlm.nih.gov/Genbank) accession numbers for phages are L5 (Z18946), D29 (AF022214), Bxb1 (AF271693), TM4 (AF068845), Barnyard (AY129339), Bxz1 (AY129337), Bxz2 (AY129332), Che8 (AY129330), Che9c (AY129333), Che9d (AY129336), Corndog (AY129335), Cjw1 (AY129331), Omega (AY129338), Rosebush (AY129334), Catera (DQ398053), Halo (DQ398042), Wildcat (DQ398052), Pipefish (DQ398049), 244 (DQ398041), Cooper (DQ398044), Llij (DQ398045), Orion (DQ398046), PMC (DQ398050), Qyrzula (DQ398048), Bethlehem (AY500153), U2 (AY500152), Che12 (DQ398043), PBI1 (DQ398047), PG1 (AF547430), and P-Lot (DQ398051).

## **Abbreviations:**

dsDNA: double-strand DNA
kbp: kilobase-pairs
